# Access to health care for migrants in French Guiana in 2022: a qualitative study of health care system actors

**DOI:** 10.3389/fpubh.2023.1185341

**Published:** 2023-10-18

**Authors:** Gabriel Brun-Rambaud, Leslie Alcouffe, Marc-Alexandre Tareau, Antoine Adenis, Nicolas Vignier

**Affiliations:** ^1^Centre d'Investigation Clinique Antilles Guyane, Inserm CIC 1424, COREVIH Guyane, Centre Hospitalier de Cayenne, Cayenne, French Guiana; ^2^Hôpital Jean Verdier, AP-HP, UFR SMBH, Hôpitaux Universitaires Paris Seine-Saint-Denis, Hôpital Avicenne, Université Sorbonne Paris Nord, Bobigny, France; ^3^IAME, Inserm UMR 1137, Université Sorbonne Paris Nord, Université Paris Cité, Paris, France; ^4^Institut Convergences et Migrations, Health and Policy Departments, Aubervilliers, France

**Keywords:** access to health care, migrants, transients and migrants, association, health mediation, community health workers, French Guiana

## Abstract

**Background:**

Access to health care is a major public health issue. The social determinants of health have a role in accessing health care and in meeting the health needs of populations. With 281 million international migrants around the world, population movements are another major issue. Migrants are particularly exposed to precariousness during their migratory journey and after their settlement. These vulnerabilities may have deleterious effects on their health status and on their social conditions. In French Guiana, 36% of the population is of foreign origin. The objective of this study is to explore the barriers and the facilitators to accessing health care for migrants in French Guiana in 2022, from the perspective of health care professionals, social workers and local NGO actors.

**Methods:**

This research is an exploratory qualitative study based on the experiential knowledge of health care professionals, social workers and local NGO actors in French Guiana. 25 semi-structured interviews were conducted with these professionals and actors between April and June 2022, using an interview guide to explore their practices, representations and beliefs of access to health care and accompaniment of migrants in their patient journey. The interviews were transcribed and analyzed using the thematic analysis method.

**Results:**

A total of 25 health care professionals, social workers and local NGO actors were included in the study. Participants highlighted that migrants are exposed to many factors hindering their access to health care in French Guiana (administrative complexity, language barriers, financial barriers, mobility issues, etc.). With the situations of great precariousness and the inadequacies of the public authorities, associative support (social, health and legal accompaniment process provided by NGOs and associations) has an important role in providing close support to migrants. Moreover, health mediation supports migrants in their social and health care journey to lead them toward empowerment. Health mediators contribute to promote a better understanding between professionals and migrants.

**Conclusion:**

In French Guiana, associative support and health mediation promote access to health care and social accompaniment for migrants. This article highlights the issues surrounding access to health care, associative support and health mediation in the Guianese context, which is marked by significant socio-cultural diversity and precariousness. Considering the benefits of associative support and health mediation, as well as social inequalities in health, is essential for health care professionals, social workers, local NGO actors, associations, public health authorities and political decision-makers to initiate concrete and suitable actions in favor of access to health care and social support for migrants in French Guiana.

## 1. Introduction

The number of international migrants worldwide is 281 million, or 3.6% of the world's population (1). In France, 10% of the population was born abroad (2). The fields of migration and health are strongly interrelated. Studies on the health of international migrants have shown that the migration route, living conditions, economic insecurity and loss of social ties in the country of arrival may have adverse effects on their health (3, 4). Health must be considered globally, as a combination of several factors. Thus, individuals must have the necessary conditions to be able to afford housing, education, adequate food, a minimum income, a stable environment, social justice and fair treatment (5).

With a specific geographical position, French Guiana, a French overseas department located in South America, is a historical territory of migration. The history of French Guiana and the successive waves of migration experienced have contributed to making its population plural. Indeed, 36% of the population was born abroad and several communities compose the Guianese population. The Haitian, Brazilian, Surinamese, Dominican and Chinese populations are notably present and based in the territory. The Surinamese lived a period of civil war in the mid-1980's, which led many people to flee their country and settle in French Guiana (6). The Republic of Haiti has been facing strong political and economic instability for several years, affecting the living conditions of Haitians (7–9). The country has experienced several natural disasters, including the 2010 earthquake, which led many Haitians to leave their country. Many Brazilians from the northern border regions have also settled in French Guiana. They are particularly represented in the illegal gold mining industry (10). Finally, more recent migrations have been observed from South American or Caribbean countries such as Venezuela, Cuba or from more distant countries such as Syria, Morocco or Yemen (11). In addition, all these communities present in French Guiana participate in the multilingualism of the territory. The languages spoken are, among others, French, six Amerindian languages, the variants of Nenge Tongo and Saamaka among the Bushinengue, Guianese Creole, but also all languages resulting from migration, Haitian Creole, West Indian Creole, Sranan Tongo, Spanish, Portuguese, English, Hmong, Arabic etc. Thus, the multiethnic and multicultural nature of French Guiana also enables the expression of a great diversity of practices, representations and beliefs related to health (12).

French Guiana is exposed to major and multiple public health issues. The territory is affected by a generalized HIV epidemic, with a prevalence rate of over 1% in the general population. The vast majority of Persons Living with HIV (PLHIV) are foreign-born (85% in 2018) (13). In 2014, among people declaring that they had already been screened for hepatitis B, 5% indicated that they had had hepatitis B (14). In 2017, the tuberculosis reporting rate was 32.5 per 100,000 population (15). Between 2007 and 2016, the incidence rate for all cancers in men was 272.2 per 100,000, per year. For women, this incidence rate was 202.9. Between 2018 and 2019, the neonatal incidence rate of sickle cell disease per 10,000 newborns tested was 61.5 (16). In 2020, the incidence of malaria was 0.55 ‰ population. For 36% of malaria attacks with a presumed place of infection located in French Guiana, the infections occurred in the forest and at gold-mining sites, particularly frequented by populations of Brazilian origin (17). In 2018, overweight and obesity affected 51% of Guianese (47% in mainland France), particularly women. This obesity favors the appearance of high blood pressure (34% of obese people) and diabetes which affects 8% of Guianese aged 15 years or more (18). Cerebrovascular accidents and cardiovascular pathologies represent one of the main causes of mortality in people under 65 years of age. In 2014, the incidence rate for stroke in French Guiana was 189.5 per 100,000 for ischemic stroke and 65.7 per 100,000 for hemorrhagic stroke (19). Regarding the fertility rate, women have an average of 3.5 children (2 children in mainland France) (20). The birth rate is the second highest of the French regions (27.5 ‰ vs. 10.7 ‰ in mainland France) (21). Finally, in 2020, life expectancy at birth in French Guiana was estimated at 80 years, it was the highest in Latin America despite major inequalities in distribution (22, 23). These social inequalities in health could be partly determined by difficulties in accessing health care.

As defined by Gulliford et al. (24), access to health care is the result of a complex interaction between health care structures, patients, the social environment (25), and the social determinants of health (26). In France, despite a universal health care system, major inequalities in access to health care have been observed (27). These inequalities may arise from various obstacles, including financial, legal, geographical, linguistic and cultural; from situations of discrimination experienced in the health care system; and from the complexity of the health care system and procedures for accessing rights (28–32). These social inequalities are less studied but could be exacerbated in the specific context of French Guiana.

**Figure 1:**
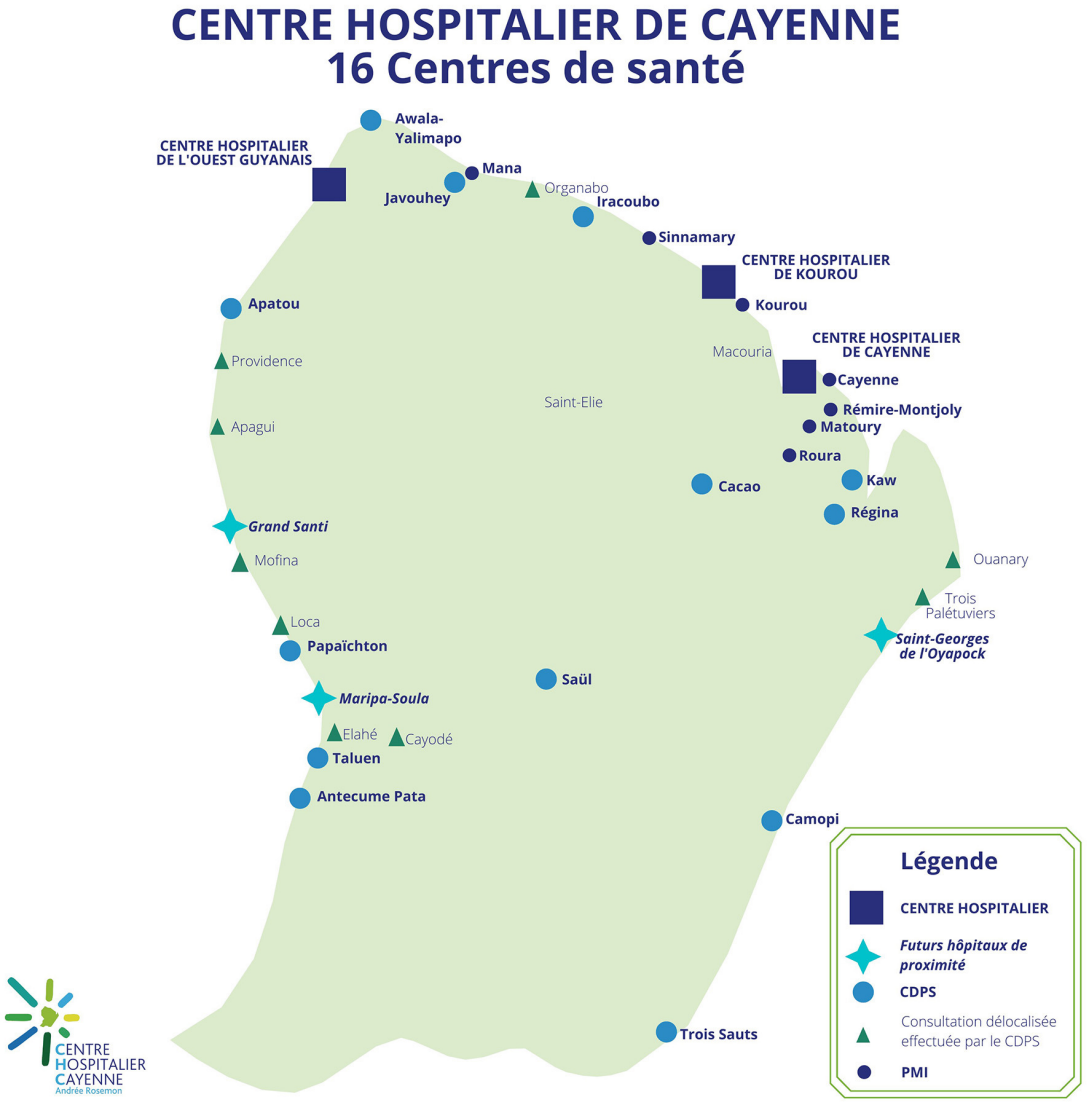
Distribution of health structures in French Guiana, GHT, 2022. Translation of the map legend, from top to bottom: *Hospitals, future local hospitals, off-site prevention and health care center (CDPS), off-site consultation provided by CDPS, mother and child protection center (PMI)*. Source: Adapted from Groupement Hospitalier de Territoire de Guyane (GHT) (38).

Indeed, French Guiana is marked by precariousness which particularly affects foreign-born people (33). In 2018, the rate of people living below the poverty line was estimated at 52.9% of the population (14% in mainland France) (34). Food precariousness has strongly affected migrant, especially during the COVID-19 pandemic (35). Residential insecurity is also present among migrants, many of them living in informal settlements (36). The surface area of French Guiana is vast (83,000 km^2^), the population is relatively small and mainly concentrated on the coast. The public transport network is poorly developed and complicates access to health care for people living in urban or rural areas (37). The health care system is based on primary health care, particularly in the isolated districts where the *Centres délocalisés de prévention et de soins* (CDPS) ensure access to health care for people living far from the coast (Figure 1). The density of health care professionals is among the lowest in France (18), and the territory suffers from a lack of health care, particularly in general and specialized medicine (39). In 2019, it was estimated that 85% of the Guianese population had used medical health care but that a third of them had had to delay or forego it (particularly for financial reasons), with foreigners foregoing more than people of French nationality (25% vs. 15%) (18, 40). In French Guiana, difficulties in accessing health care are particularly present even though the population expresses significant health needs, particularly regarding infectious diseases, metabolic diseases and sexual and reproductive health.

Regular or irregular migrants may benefit from different health assistance in French Guiana. Regarding the regular migrants, the French health insurance entitles anyone working or residing in France on a regular and stable basis to have their health care costs covered, on a personal basis and continuously throughout their life (41). There is also a specific French procedure for issuing residence permits for health reasons to foreigners if they are in “*a health status requiring medical care, the lack of which could have exceptionally serious consequences for them*,” and if they are unable to access appropriate treatment and health care in their country of origin (42). Regarding the irregular migrants, the *Aide médicale d'État* (AME) is a system that allows foreigners who are in France in an irregular situation for more than 3 months to have access to health care. The AME is granted on the basis of residence and resources, is delivered for 1 year and must be renewed each year. The AME provides 100% reimbursement of health care costs (43). The urgent and vital care system is for people who have been in France for <3months, or who are waiting for their AME. The system guarantees health care whose absence “*would be life-threatening or could lead to a serious and lasting deterioration in the health status of the person or the unborn child*” (44). Finally, the *Permanences d'accès aux soins de santé* (PASS) are dedicated medical and social health care services for people in precarious situations and most often without health coverage. PASS help these people to access the French health care system and obtain recognition of their rights, as well as making the transition to the mainstream health care system (45).

Public authorities have a major responsibility to respond to public health issues in order to enable all individuals, without distinction, to enjoy the highest attainable standard of health. Migration issues are also crucial to public health. Migrants may be confronted with situations of emergency, precariousness and vulnerability that affect their health status (malnutrition, infectious diseases, trauma, etc.). The right to health, enshrined in the WHO Constitution, is a fundamental right that must be respected (46). Barriers to access to health care for migrants must be limited or eliminated. Innovative measures, such as health mediation, enable migrants to be supported in their social and health care journey. Such measures are essential to ensure the accessibility, stability and sustainability of the health care system. Research and quantitative, qualitative or mixed studies may help to provide a better understanding of these issues, particularly for public health authorities and political decision-makers. These dimensions have been insufficiently studied in French Guiana.

In this context, we hypothesize that the health care offer in French Guiana is insufficient regarding the needs and demands of the population in terms of health; that migrants are particularly affected by a set of barriers hindering their access to health rights and health care in French Guiana; that migrants live in a situation of high socio-economic insecurity having deleterious consequences on their general health. In very precarious situations, health concerns may be secondary to housing and food issues. However, access to health care, health coverage and appropriate social and health care support may help reduce social inequalities in health, particularly by reducing financial barriers.

Despite the conceptualization of accessibility in health (25), there is a lack of literature about access to health care. In French Guiana, some epidemiological studies have highlighted public health issues in the territory (13, 40, 47). Some qualitative studies have also been conducted, particularly on access to health care for migrants and discrimination in health (48–52). However, little is known regarding the importance of health mediation (provided by community health workers) and associative support in the social and health care journey of patients, especially migrants. To address this gap, our qualitative study aims to generate new evidence on access to health care for migrants in French Guiana and the barriers they face.

The objective of this study is to explore the barriers and the facilitators to accessing health care for migrants in French Guiana in 2022, from the perspective of health care professionals, social workers and local NGO actors.

## 2. Methods

### 2.1. Study population

This research is an exploratory cross-sectional qualitative study based on the experiential knowledge of health care professionals, social workers and local NGO actors in French Guiana in 2022. It aims to identify and understand complex and contextual phenomena. The study is based on phenomenology, which enables us to understand the meaning of subjective phenomena using participants' narratives to explore and describe the meaning given to a specific experience.

The study population is composed of health care professionals, social workers and NGO local actors working in the main cities of French Guiana (Cayenne, Kourou, Saint-Laurent-du-Maroni, and Saint-Georges-de-l'Oyapock), and in the CDPSs of the isolated districts within the territory.

The population of interest refers to migrants, understood as any person born abroad, of foreign nationality and residing stably in French Guiana. The term “migrants” thus refers to all persons, whether or not they have a residence permit, whether they are refugees, undocumented or seeking asylum (53).

*Médiation en santé* and *médiateur en santé* are terms used in the French context and applicable in French Guiana. They refer to facilitating access to rights, prevention and care for vulnerable people distanced from the health care system (54). In this study, we decided to use these terms and translate them respectively, as *health mediation* and *health mediator* (55).

### 2.2. Sampling and recruitment of participants

In total, 44 persons were invited to participate in the study, 25 accepted and 19 declined. The reasons for declining were: invalid contact information, no reply despite reminders, not working with migrants, recent arrival in French Guiana, on professional or personal travel, unavailable. Participants were recruited either during spontaneous or organized encounters, or by sending an email using contact details available online or provided by health care and prevention networks. Thus, participants were selected using a non-probability and convenience sample. The sample size was determined by the data saturation that emerged as the interviews progressed. Recruitment of participants ceased after 25 inclusions, as sufficient data were collected and data saturation was reached. The inclusion criteria were as follows: participants must work as health care professionals, social workers or local NGO actors, they must have experience of working with migrants living in French Guiana, and they must agree to participate in the study.

### 2.3. Data collection

The data were collected during 45-min semi-structured interviews, conducted face-to-face in a confidential setting. They were conducted by GBR having a background in political sciences and public health and by MAT having a background in anthropology and ethnobotany and extensive experience in qualitative studies. An interview guide was developed and piloted prior to the study. It was composed of two parts: the professionals' and actors' representations of access to health care for migrants in French Guiana, and the professionals' and actors' practices concerning the support of migrants in their patient journey.

### 2.4. Data analysis

Based on the approach of Levesque et al. (25), our analytical framework follows the conceptualization of access to health care along several dimensions: approachability, availability, accessibility, acceptability, quality, equality and equity. According to this approach, access to health care is determined by a complex interaction between the health care system, the characteristics of the individuals and the social environment. The main factors of access to health care explained in this study have already been studied at the international level, particularly in the case of migrants living in Europe or South America, which provides a robust basis for this analytical framework. Our conceptual framework is also based on the social determinants of health as defined by the World Health Organization (26). Thus, the health status of populations may depend on many determinants such as individual characteristics, living environments, systems (education, health care, urban planning) and contexts (economic, social, cultural, political, environmental) that evolve within a defined spatio-temporal framework. This overall combination of factors may have a positive or negative impact on the health of populations. Health and access to health care are thus described and analyzed as part of a social and health care system influenced by the social determinants of health.

For this study, we used the thematic analysis method to extract key themes from the interviews in order to describe, report and analyze the participants' viewpoints. We started by familiarizing ourselves with the data collected by listening to the audio recordings and reading the transcripts. We continued the analysis by selecting verbatims and assigning them a code in the data analysis software. We worked with the MAXQDA Plus 2022^®^ software (Release 22.2.0). Following an iterative approach, some verbatims were recoded to enable recurring themes to be grouped together and to ensure coherence within our code matrix. The analysis' structuring continued with the interpretation of the results around several themes. Interpretation was based on the knowledge available in the literature, to propose explanations and comparisons.

Interviews were recorded with the participants' consent. They were conducted and fully transcribed in French. Verbatims used in the article were translated into English by the main author, respecting the participants' statements as faithfully as possible, and were checked by the co-authors. The socio-demographic characteristics of the participants were recorded in a spreadsheet and described in median and percentage with the help of R^®^ software.

Different techniques were used to ensure rigor and trustworthiness of data analysis. Data saturation was reached when participants reported similar experiences, when no new themes emerged during the interviews, and when data collected could be analyzed to both group and single out experiences. Audio recordings, transcripts and interpretation of results were verified by the co-authors (public health and anthropologist researchers). Interpretation of results was associated with the literature to illustrate or challenge article's results. Regarding data triangulation, our field observations have also contributed to highlight or question our interpretation of results.

### 2.5. Ethical considerations

The study was authorized by the *Comité de protection des personnes* (CPP) Sud-Est I under CPP number 2021-119 and its substantial modification CPP 2021-119MS02 as a qualitative ancillary survey for the *Parcours d'Haïti* project. During each interview, the objectives and conditions of the study were explained to the participants and an information note was given to them. The participants' non-opposition to the study was collected as well as their agreement to the recording of the interview. A unique and confidential number was assigned to each participant. All interviews were anonymized.

## 3. Results

### 3.1. Socio-demographic data of participants

A total of 25 interviews were conducted between April and June 2022 with 8 men (32%) and 17 women (68%). The median age of the participants interviewed was 38 years with an interquartile range of 10 years. In terms of territorial distribution, 13 (52%) practiced in Cayenne, 5 (20%) in Kourou, 4 (16%) in Saint-Laurent-du-Maroni (a western border town), and 3 (12%) in Saint-Georges (an eastern border town). 12 (48%) of the participants worked as physicians or midwives, either as private practitioners or as employees, 6 (24%) as nurses, psychologists or health care managers, either as private practitioners or as employees, and 7 (28%) as social workers or health mediators.

### 3.2. Main barriers and facilitators to access to health care for migrants

**Figure 2:**
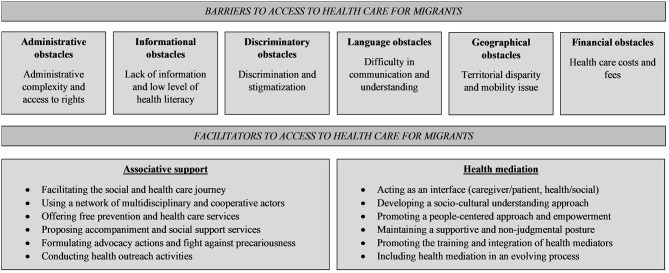
Diagram of the main barriers and facilitators to access to health care for migrants reported by health care professionals, social workers and local NGO actors in French Guiana in 2022.

The main barriers and facilitators to access to health care for migrants reported by health care professionals, social workers and local NGO actors are illustrated in Figure 2.

### 3.3. Barriers to access to health care

#### 3.3.1. Administrative complexities in accessing the health care system

Participants detailed the complexity of administrative procedures in the health care journey for migrants. In France, whether they are in a regular or irregular situation, migrants may apply for specific health coverage from the health insurance system. When preparing applications for health coverage or social assistance, several documents are required, some have been described as abusive by participants (birth certificate, certificate of accommodation, etc.). They also reported long waiting times before migrants could consult health care professionals or obtain health coverage. These complexities were described as affecting both the professionals in their support work and the migrants in their social and health care journey.

“*There are some, as soon as they have filed two, three times, and that they don't get a return, they stay like that. [...] and sometimes there wasn't much missing to get the health insurance file complete. It's just an explanation on how to fill that out that they didn't have, so they suffer, they suffer” (Participant 6, local NGO actor)*.

#### 3.3.2. Health information and literacy

Participants mentioned the difficulties in accessing information. They declared that, for users, the lack or absence of information can make it difficult for them to understand the health care system and to find their way around the various health care services. Some participants recognized a lack of access to information on existing health and social services, which could help them to better orient and refer migrants. Participants also underlined a variable level of health literacy^1^ between people which may lead to difficulties in explaining the patient journey or the care needed in some situations. They reported the necessity for migrants to be fully involved in and make decisions about their social and health care journey. According to them, this requires, among other things, appropriate support, a good level of understanding of the health care system and empowerment. The health mediators reminded the importance of their role in ensuring orientation in the health care system and understanding of care.

“*And as health mediators, it is our job to pass on the right information so that the person can take a stand and seek treatment with full understanding of the situation” (P13, local NGO actor)*.

#### 3.3.3. Linguistic barriers

Related to health literacy, the language barrier is a major problem for the participants. It hinders a clear and reciprocal exchange between health and social professionals, local NGO actors and migrants on individual health and social issues. Despite the interest of participants in understanding and learning the foreign languages spoken by migrants, the lack of vocabulary remains an obstacle to understanding the health care journey. In the multilingual context of French Guiana, participants are looking for health mediators and interpreters to help them dealing with the linguistic difficulties they encounter. To overcome the language barrier, digital tools, such as online automatic translation, may be used by health care professionals, social workers and local NGO actors to exchange during consultations and accompaniments. But these solutions remain limited and temporary according to them.

“*When you speak four words like me in a little bit of every language, you can see that you can't get to the bottom of things. People are frustrated and so are we” (P2, health care professional)*.

#### 3.3.4. Discrimination et stigmatization

Participants mentioned the discriminatory and stigmatizing representations and attitudes that may be exercised against migrants. Local NGO actors and associations are often witnesses or spokespersons for situations of discrimination, particularly within health and social facilities. The Haitian community seems to be particularly affected by this daily discrimination, as well as the LGBTQIA+ community. Participants report that these situations may be experienced and defined as violence in the health care journey. In addition to discrimination and marginalization related to origin and social status, stigmatization related to health status is also present. In the case of highly stigmatized diseases such as HIV/AIDS, some PLHIV prefer not to inform the people around them of their serological status. Confidentiality is thus perceived as a very important element in the accompaniment.

“*And so the lady, she gave birth (at the hospital) and there was one person (from the team) who was like “yeah you see, it's the government's fault, it's going to be one more French person*^2^”* (P22, health care professional)*.

#### 3.3.5. Financial barriers

Many participants identified financial barriers as an obstacle to accessing health care for migrants. These barriers are often correlated with the high level of uncertainty and insecurity in which migrants live daily. Precariousness leads some migrants to delay their access to health care, to put their health in second place, by order of priority of budget and daily survival. Medical fees, outside of social protection, are most of the time difficult to afford and to consider for migrants who may already have difficulties to meet their basic needs. Participants reported the priority needs expressed by migrants, particularly primary needs such as food, housing, working and having documents. These conditions are prioritized over health and may lead people not seeking health care. Financial barriers affect the entire health and social system and despite the presence of free health care facilities, the expenses associated with care may remain very high.

“*When you don't have social rights open and you want to have access to liberal health care, you have to pay. But paying twenty euros for a health consultation, well…, you have to take out these twenty euros” (P14, health care professional)*.

#### 3.3.6. Mobility issues

Participants raised the important issue of mobility and access to health care facilities. According to them, public transport is insufficient and the itineraries do not serve certain isolated districts, coastal districts or remote and/or precarious urban housing areas. Alternative means of mobility exist such as collective cabs, clandestine cabs, pirogues on the Guianese rivers or airplanes but they are very expensive. Participants also pointed out that some small towns, such as Kourou or Saint-Georges, are less affected by the mobility issue and the problem of long and expensive trips. Furthermore, with the restrictions on movement to which migrants are exposed, especially those in an irregular situation, fear of the police and of arrest is one of the barriers frequently mentioned by participants.

“*We don't realize what happens beforehand, the difficulty of coming, physically, of coming on foot, from very far away, of respecting schedules, because the transportation doesn't pass at the right times, or because we don't have the money to take the bus anymore. All these things we don't have any idea of that” (P2, health care professional)*.

### 3.4. “*I guide them toward the solution*”: the importance of associative support in the social and health care journey

#### 3.4.1. Facilitating the social and health care journey

The associative support is defined as a process of social, health and legal support carried out by local NGOs and associations acting within the framework of a common project in favor of human rights. Local NGO actors and associations accompany and guide migrants within the health care system and provide them with support in drawing up administrative and social files to assert their rights. Some participants emphasize the importance of the support provided by local NGOs, which makes it possible to fill a gap in the state system and to offer migrants a range of prevention and care services adapted to their needs. Others recognize that the complexity of the health care journey may lead migrants to live in isolation or wander until they meet associations or NGOs that may respond to their social and health concerns.

“*We accompany them in terms of access to health care, rights, residence permits, food packages, setting up a home address, obtaining a financial resource to have stamps for residence permits, that's it. We really have this administrative, social and physical accompaniment to appointments as well” (P20, local NGO actor)*.

#### 3.4.2. A supportive network of actors

According to the participants, in French Guiana, relations between health care professionals, social workers and local NGO actors are taking place in a context of insufficient supply compared to demand. According to them, this situation leads to the strengthening of cooperation in order to allow for the effective orientation of migrants in terms of care or social support. These collaborations are described as being consolidated thanks to a multidisciplinary, active and supportive network and to a common sharing of values, a state of mind, and even a militancy. Participants stressed the importance of facilitating the patient journey as much as possible for migrants who are going through a complex life course. The relationship of trust between professionals and migrants is also an element that was put forward and perceived as essential to facilitate access, consent and adherence to health care.

“*It's a job to put people at ease and to manage, almost like a radio, to be on the frequency that will make the person feel as comfortable as possible, but it doesn't always work. [...] they always have the right to say no, not to say everything, to say it at their own pace” (P15, local NGO actor)*.

#### 3.4.3. The health outreach approach

Participants mentioned the importance of the health outreach approach, which aims to reach out to migrants to enable them to access health care and social support. Many local NGOs offer health outreach, prevention and/or care services, using off-site facilities such as mobile clinics that travel to districts where migrants live, particularly in informal settlements. Participants noted that this proximity facilitates access to prevention, in particular through the existence of a culture of health prevention that is developed among health actors and through the associative network being at the origin of multiple health projects and actions.

“*We also do outreach, we go to the people, it's the idea of “going to the people” that we talk about a lot today [...] that's what's going to make it easier to hook people” (P9, local NGO actor).*

#### 3.4.4. Free health care services

Many participants identified financial barriers as an obstacle to accessing health care for migrants. These barriers are often correlated with the high level of precariousness in which migrants live daily. Obtaining health coverage, such as the AME, allows beneficiaries not to advance health care costs and to be refunded up to 100% for a set of care defined by the health insurance. Participants emphasized that for people who do not yet have health coverage, free health care services exist in French Guiana, such as the mobile clinics of several local NGOs, the PASS, the *Protection maternelle et infantile* (PMI) or the CDPS. The free and unconditional nature of these services helps to overcome some of the financial barriers to which migrants may be exposed.

“*Sometimes there are patients who come for the first time, and if they don't have money, we'll refer them, to go to the Red Cross, the PASS, to the facilities where they can get free care” (P21, health care professional).*

### 3.5. “*We are here to connect*”: the health mediation

#### 3.5.1. Health mediation as an interface

Many of the participants emphasized the interest and importance of the role of health mediators in the social and health care journey of migrants. In a context of low health literacy and distinct representations of the body and health, health mediators play a role in linking structures and individuals. The link is linguistic, cultural and technical. Health mediators promote the link between professionals and migrants, between health and social issues, between the needs expressed and the means available. Health mediators may also organize workshops such as discussion groups, expression groups or information groups for migrants.

“*The work of health mediation is very important, because we act as an interface between the person who is vulnerable and the administrations, the institutions, the health professionals, who do not necessarily have the necessary tact to talk to people. And our role as health mediators is precisely to be able to bridge these two worlds” (P13, local NGO actor)*.

#### 3.5.2. Cultural understanding

In the multicultural context of French Guiana, participants saw cultural understanding as a central issue. They highlighted the importance of having knowledge about the social, cultural and traditional issues of the different communities. They emphasized that migrants may benefit from listening to health mediators, especially about their health needs, and that they may also share their life stories. Participants noted that exchanges may be facilitated when the health mediators share a culture, a language or a pathology with the migrants. Moreover, they insisted on the importance of traditional medicines and medicinal-magical practices in the care of migrants in French Guiana. Some participants admitted that they were not very familiar with these cultural and medicinal practices and that they did not inform themselves sufficiently with users.

“*The cultural specificities are completely different, so the representations of health, of treatment, everything is different. To really access people, make a connection, it's..., you have to take that into account” (P9, local NGO actor)*,

#### 3.5.3. Role confusion

Participants stressed the confusion that exists between the different roles of health mediators, interpreters and translators. Health care professionals, social workers and local NGO actors usually rely on health mediators to take on the role of interpreter and thus facilitate understanding in the context of a medical consultation or social support. However, the health mediators specified that interpreting is not their main mission and that they are not all trained to do it. They added that their activity is not limited to interpreting and translation.

“*It's not our role to translate what the physician says, so often physicians tend to think of us as translators, but we put the framework back, “we don't do translation, we are health mediators” (P13, local NGO actor)*.

#### 3.5.4. Health mediators: a profession under pressure

Finally, participants highlighted the lack of infrastructure and staff in French Guiana. Faced with the demand, these inadequacies may lead health mediators to undergo a significant workload, whether they are salaried or volunteers. Participants emphasized that this workload may lead to professional exhaustion, which has consequences on the work of accompaniment as well as on the personal life of the health mediators, particularly in terms of mental health. Their listening position as well as the complex life stories reported by the migrants may expose them to vicarious traumas. Moreover, health mediators often witness discrimination experienced by migrants in their daily lives as well as in health and social structures. Health mediators, especially of foreign origin, may also be personally exposed to discriminatory attitudes.

“*It happens that people come to see our teams at home, up to twenty-one, twenty-two o'clock, in their private and family lives, to ask questions. So the professional posture is complicated, making the difference between professional life and private life” (P25, local NGO actor)*.

## 4. Discussion

In French Guiana, migrants are exposed to many factors that hinder their access to health care (administrative complexity, language barriers, financial barriers, mobility issues, etc.). Health care professionals, social workers and local NGO actors form a multidisciplinary network which helps migrants navigate their social and health care journey. Faced with the situations of great precariousness and with the inadequacies of public authorities, local NGOs have an important role in the accompaniment of migrants. Health mediation, acting as a real interface, is essential in supporting migrants in their social and health care journey to lead them toward empowerment. Health mediators promote a better understanding between professionals and migrants. Tensions, workload, discrimination and the lack of definition of the profession's missions may jeopardize the work of health mediators.

### 4.1. General discussion

Regarding the complexity of the social and health care journey of migrants, the French Guiana 2021 report of the French Defender of Rights highlighted the inadequacies of public services in the territory, with complaints concerning foreigners' rights (12% of referrals) as well as social protection and social security (7% of referrals) (58). With the complexity of the procedures, non-use of services is a loss of opportunity to obtain health coverage and thus to access prevention and health care. The damaging effects of delays and restrictions in health coverage on migrants' health and public health have been described (50). Our study confirms that health mediators have a major role in helping migrants overcome administrative and access to rights difficulties, as in the context of applying for health coverage for example (31). Our study highlights lack of information as well as language barriers as elements that hinder access to health care for migrants. Origlia Ikhilor et al. and Kuan et al. (59, 60) have detailed situations where language barriers may lead to misunderstandings or misconceptions about the patient journey. Mestre (61) has described the importance of understanding as well as patient consent to fight against institutional violence in health care system. Thus, health mediators help to facilitate interactions and reinforce understanding of the health care system among the people they accompany. Although their role is not limited to interpreting, they can mitigate or even eliminate the language barrier. Several studies have described the aptitudes that health mediators may have regarding understanding the social, cultural and traditional issues of different communities (62–64). Our study also confirms that this in-depth knowledge of contexts may be used to better support migrants. Tareau et al. (65) have shown that traditional medicines and medicinal-magical practices are very present in French Guiana. Gil-González et al. (66) explained that barriers to access to health care could be accentuated when health care professionals, social workers and local NGO actors were not able to understand, explore and accept social and cultural differences. Verrept H. detailed cultural understanding in health as a factor facilitating the achievement of the highest level of health of individuals (62). It appears that the support work of health mediators in French Guiana helps to consider health as a system that promote cultural diversity, prevent exclusion, and support the empowerment and integration of migrants into society. While they have been observed and studied on many occasions in a Guianese or international context (48, 63, 67, 68), discriminatory attitudes toward migrants have also been reported in our study. They constitute a major obstacle to access to health care and have deleterious effects on the social and health care journey and integration process of migrants. Through his work, Leduc (69) has shown the tensions and even discrimination that may exist with health insurance services, particularly when it concerns granting migrants their health care rights. Our study reports similar situations of discrimination within the social, health and access to rights facilities. Contrary to the findings of Santalahti et al. (70), our study does not show a generalized distrust of social and health facilities by migrants. Through their support, their advocacy and their militant actions, certain local NGOs and associations may be identified as relays for support, referral and even the fight against discrimination and stigmatization.

Regarding the associative support, our study confirms a tendency to delegate public service missions to local NGOs and associations in French Guiana. This delegation may be carried out within an official framework, but it may also exist *de facto*, in a palliative context of the inadequacies of the public authorities. Considering the importance and persistence of social inequalities in health, reducing them remains a major public health priority for which the state is responsible (71). In French Guiana, however, certain public service missions are shifted to local NGOs, particularly in the areas of prevention, care and support for vulnerable populations, including migrants. To fight against marginalization, health care and social support for migrants should be provided within the framework of common law (72). Moreover, to ensure that health mediation is not part of a palliative approach to a dysfunctional health care system, the approach of the public authorities should be strengthened with regard to the responsibilities assumed by NGOs and associations in French Guiana in the welcoming and accompanying of vulnerable populations and the fighting against social inequalities in health (55). Lebano et al. (26) have described the lack of coordination between actors as a factor in the loss of opportunity in access to health care for migrants. In French Guiana, the specific and even complex geography of the territory and the low density of health professionals seem to lead all the social and health actors to necessarily interact with each other and to set up partnerships and cooperation that are beneficial for the support, accompaniment and referral of migrants. Moreover, cross-border cooperation in health with Suriname and Brazil is another facilitator, particularly regarding access to health care for migrants and/or cross-border workers living with specific diseases such as HIV/AIDS (73, 74). Thus, the local NGO actors are essential relays for migrants to meet the needs for orientation, access to rights, prevention and care, and therefore for health mediation. Thanks to a dynamic and cooperative network, local NGO actors may also provide social and psychological support, in a holistic approach to health. Arnold et al. and Steinbrook et al. (75, 76) have shown that the distance between a home and a health care facility, as well as the lack of or inadequate transport, were barriers to access to health care for migrants. Our study highlights territorial disparities and mobility as major issues in French Guiana. Indeed, isolated districts have fewer social and health care facilities, NGOs, and medical specialists than coastal towns. For some isolated districts, the theoretical time for accessing health care is more than 1 h of transport on average (77). Thus, migrants may face mobility difficulties, due to the lack of public transport, the costs of travel or even the fear of police checks. As Popovici et al. and Adloff (78, 79) have shown, our study also underlines the importance of setting up off-site actions and deploying an health outreach approach in order to overcome mobility-related obstacles. Moreover, outreach actions and workshops, such as discussion groups, help to address prevention and care issues by reducing the geographical, social or symbolic distance that sometimes exists between migrants and health and social professionals (80). Thus, it seems necessary to strengthen support and health mediation measures in a health outreach approach to better support vulnerable populations in their social and health care journey and to promote their individual and/or collective empowerment, allowing “*greater control over decisions and actions that affect their health*” (81, 82). Financial barriers are a major obstacle to accessing health care. Health care expenses may affect the budgets of migrants and families who are experiencing financial difficulties. Mooney G.H. indicated that health care expenses also include travel, transportation, housing and may vary greatly between people, especially depending on the distance to a health facility (24). Moreover, the living conditions, housing, food and employment of migrants may have deleterious short- and long-term effects on their health status (51). Gosselin A. *et al*. described the predominance of residential and administrative precariousness to which migrants were exposed from the moment they arrived in France and for several years (83). Our study shows the decisive role played by local NGOs and associations when they accompany or refer migrants to free health care services (mobile clinics, PASS, AME). These measures help to overcome certain financial barriers by offering migrants the possibility of receiving free care unconditionally, regardless of their status. In a global approach to health, acting on the precariousness that migrants experience daily should be a primary necessity in order to guarantee them the best health condition and wellbeing.

Regarding the health mediation, patient associations, carers and health mediators are perceived as important levers for giving voice to and empowering people living with a disease or in vulnerable situations (84). Complementing the associative support, health mediation is a pivotal approach. Health mediation seems to be particularly relevant to the multicultural context of French Guiana. By deploying health mediators to assist migrants, health mediation may simultaneously overcome several barriers to access to health care for migrants. Without a health mediation approach, migrants' difficulties in accessing health care may be exacerbated. But health mediation faces certain difficulties. It is not yet widely implemented, and is still struggling to be established, despite the benefits it may offer to migrants in their social and health care journey. Concerned by their social and health care journey and aware of their own needs, the patients or the migrants are able to position themselves as “social reformers” (85, 86). This enables them to act to transform health representations and practices as well as their relations with health and social professionals. As part of the concept of health democracy—in which professionals, users and political decision-makers act together in the field of public health policies—health mediation helps to remove certain obstacles that may have an influence in the health care renunciation (87). Moreover, health mediation is based on the main principles of health outreach, social support and empowerment. Indeed, the role of health mediation is not to replace people and do everything for them, but rather to make them autonomous in their decisions so that they are fully aware of and active in their social and health care journey. Health care professionals, social workers and local NGO actors in French Guiana agree on the need for more health mediation in the health care journey and social support of migrants. The publication of a new health law in 2016 and the health mediation competency reference framework in 2017 in France allowed for a real advance regarding the structuring and recognition of the profession (88). However, this recognition has not been followed by a formal status and the creation of jobs (89). These inadequacies block the emergence of health mediation as an essential measure for access to health care, particularly for migrants. In addition, Kühlbrandt (90) has described the professional insecurity to which health mediators are exposed. Indeed, health mediators mainly work in NGOs or associations, with a status varying from voluntary work to paid employment. Contracts are often of short duration, financed through calls for projects limited in time, leading to an unsecure career path. Moreover, salaries are generally low and dependent on public or private subsidies, which are randomly renewed, thus reinforcing the precarious nature of the profession (55, 62). These elements have real consequences on the general perception of the profession, on the turnover rate, on the isolation of health mediators and even on their mental health (80). The development of health mediation should require full integration into the health care system and a decompartmentalization between health mediators and health and social professionals (55). Moreover, the implementation of national strategies to support and recognize health mediation and its professionalization is wanted by the health care professionals, social workers and local NGO actors who have experience such collaborations. Several initiatives have demonstrated the effectiveness of health mediation in a holistic approach to the health of vulnerable populations (89, 91). The originality of health mediation is characterized by the interstitial area it covers between the medical and social fields, and by the place it confers to the social determinants of health in the support of vulnerable populations (55, 80). At the junction of health and social fields, the functions and missions of health mediation are numerous and have a power of innovation aiming to improve the social and health care journey of the people accompanied and to promote their empowerment (80). Since 2018, the University of French Guiana and the Sorbonne Paris Nord University offer a university diploma in health mediation (92). In France, the offer of health mediation training courses has recently been expanded, enabling the profiles of people with or without experience in this field to be targeted (54). However, Hashar-Noé et al. (71) have shown that the training offer in France was unbalanced between the different territories, that the teaching provided lacked uniformity and that the hourly volumes were not all equivalent. To improve the working conditions of health mediators and to support migrants in their social and health care journey, the importance of setting up moments for analysis of professional practices was stressed. Meetings between health mediation workers and psychological support are also moments of reflection to be encouraged for health mediators (62, 89). Indeed, the professional, emotional or allostatic load that are experienced during accompaniment may lead to professional exhaustion, to an anxiety-depression syndrome or to vicarious traumas. Finally, the institutionalization of health mediation is often conditional on cost-benefit analysis, particularly by public authorities (85). Full recognition also depends on greater accessibility of training courses (professional and diploma courses) as well as on a durability of mediation contracts within social, health and associative structures.

### 4.2. Recommendations

Based on the results of our research and in order to improve access to health care for migrants in French Guiana, we propose some recommendations for policy and practice changes. These recommendations are based on interviews, data analysis and conclusions of this article. They are formulated to health care professionals, social workers and local NGO actors, and particularly to public health authorities and political decision-makers.

- Regarding the health mediation: developing training (initial and lifelong learning) and diplomas in health mediation and interpreting; proposing a plan to hire and finance these professionals in social and health facilities to better support migrants in their social and health care journey in French Guiana; strengthening the institutional recognition of health mediation and health mediator.
- Regarding the health outreach approach: strengthening local initiatives through a health outreach approach in priority districts where migrants live; ensuring accessibility to social and health facilities through an adapted and affordable transport network (free public transport for health reasons for example); guaranteeing free access to health care for migrants in an irregular situation and/or without health coverage in health facilities.
- Regarding the recruitment's policy: encouraging the recruitment of local professionals and applying strategic choices limiting staff turnover; offering sensitization sessions on the different cultures and medicinal practices of French Guiana to health care professionals, social workers and local NGO actors.
- Regarding the language barriers: implementing and ensuring access to a regional professional health care interpreting service and strengthening the presence of health mediators in social and health facilities; developing information and prevention content adapted to cultural contexts, health literacy and languages spoken in French Guiana; adapting the information and orientation signs in social and health facilities in several languages and images.
- Regarding the information and the coordination: launching an information campaign on the health care system in French Guiana and on access to rights for migrants; strengthening patient information and referral networks between health care professionals, social workers and local NGO actors, particularly in terms of mental health.
- Regarding the psychological support: strengthening and developing mental health services, screening and care for migrants suffering from psychological disorders, particularly post-traumatic stress.

### 4.3. Limitations and strengths

Our study does not directly report the opinions of migrants. We chose to collect only the representations and perceptions of health care professionals, social workers and local NGO actors. However, migrants could mention other barriers and have other representations, particularly concerning the contribution of associative support and health mediation. Furthermore, following the concept of *foreign gaze* (93), we recognize that local professionals and specialists are key actors in providing answers to local issues; and that our position as a researcher external to the field of study may be a limit to understanding the Guianese context *in extenso*. The study refers to a population as diverse and complex as migrants. We defined as “migrant,” any person born abroad, of foreign nationality and living currently in French Guiana. We would like to emphasize that this terminology embraces a diversity of groups, communities and individuals. Thus, further research could be conducted to explore practices and representations on access to health care from the specific viewpoints of the migrants themselves and of certain migrant communities (Brazilians, Haitians, Surinamese etc.). Moreover, we are aware that the study also refers to heterogeneous characteristics of profession and provenance. As a qualitative study that does not seek to be representative, this heterogeneity helps to explore the different practices, representations and beliefs of access to healthcare for migrants in French Guiana. We recognize that it would be interesting to pursue further studies exploring and analyzing these aspects in depth across a particular group of professionals or a particular geographical situation. For this study, we did not interview the public health authorities, administrative and political actors from French Guiana, who were nevertheless mentioned many times during our interviews. Further research could focus on the representations they may have regarding access to health care for migrants. Finally, further research could be conducted to explore more deeply mental health or health outreach initiatives as these important themes were mentioned during the interviews.

Our research is one of the first qualitative studies to focus on access to health care for migrants in French Guiana, from the perspective of experienced professionals. While studies on this topic have been mostly quantitative, our work offered the opportunity to health care professionals, social workers and local NGO actors in French Guiana to share their experiences, feelings and opinions through in-depth interviews. A strength of the study is to place health mediation as an important scientific topic in terms of access to the health care system for vulnerable populations. Although health mediation is still only partially recognized by institutions, and its position is not well known, this study shows that it is both innovative and has been established for several decades in various forms. The study took place in different cities in French Guiana (including the three main ones) and with several health actors. Professionals working in isolated facilities were also included. This diversity enriched our exploratory research and made our analysis denser. This study presents different approaches to health care and proposes support measures that allow to question the health care system, its challenges, its advantages, its evolution and its sustainability. The availability and enthusiasm of participants, who were often busy, underlines the importance of the topic and their interest in sharing their observations and proposals for improvement. Moreover, in the diverse context of French Guiana, we believe that the authors' backgrounds in public health, political science, infectiology, pharmacy, anthropology and ethnobotany contribute to enriching the study and understanding of the many issues involved in access to health care for migrants. Finally, the results and recommendations of our study provide scientific material of interest to health care professionals, social workers, local NGO actors, associations, public health authorities and political decision-makers, which may be used to structure public health responses in French Guiana.

## 5. Conclusion

The results of this study suggest that, although access to health care for migrants is subject to many barriers, associative support and health mediation are essential measures to enable them to access health care and benefit from social accompaniment. Social and health support for migrants responds to multiple issues that we tried to describe in this article. In French Guiana, local NGOs are networked and work as close as possible to the populations to provide adequate, equitable and effective support. The existence of many barriers to access to health care as well as the inadequacies of the public authorities lead local NGO actors and associations to assume *de facto* an important part of the support missions for vulnerable populations. Despite a lack of recognition of the work of health mediation, we are observing an institutionalization of the sector, with an increasing use of health mediators, especially by local NGOs. Health mediators are key players in the social and health care journey of migrants. They contribute to put migrants at the center of the accompaniment; they support the process of access to rights and health care; they facilitate the understanding of the health care system and play a major role in migrants' empowerment. This qualitative study highlights the reflections and debates surrounding access to health care, associative support and health mediation in a context marked by social and cultural diversity in French Guiana. Considering the benefits of associative support and health mediation, as well as social inequalities in health, is essential for health care professionals, social workers, local NGO actors, associations, public health authorities and political decision-makers to initiate concrete and suitable actions in favor of access to health care and social support for migrants in French Guiana.

## Data availability statement

The original contributions presented in the study are included in the article/Supplementary material, further inquiries can be directed to the corresponding author.

## Author contributions

GB-R led the overall study, wrote the interview guide, conducted the data collection, the interviews, the data analysis, and wrote the manuscript. LA contributed to the study design, the data analysis, and the manuscript edits. M-AT contributed to the interviews and the manuscript edits. AA contributed to the scientific follow-up of the research. NV supervised the overall study, contributed to the study design, the data analysis, and the manuscript edits. All authors contributed to the research design, read, reviewed, and approved the final manuscript.

## Footnotes

^1^Health literacy is defined as “*the motivation and skills of individuals to access, understand, evaluate and use information to make decisions about their health*” (56).

^2^There are several ways to obtain French nationality, and they are all very strict. For example, French nationality can be granted “*at the age of majority when a child is born in France to two foreign parents. To obtain it, the child must be resident in France at the age of 18 and have been ordinarily resident in France for a continuous or discontinuous period of at least five years since the age of 11*” (57).
