# Supplementary material for: Access to health care for migrants in French Guiana in 2022: a qualitative study of health care system actors
Source: Front Public Health. 2023 Oct 18;11:1185341. doi: 10.3389/fpubh.2023.1185341 (PMC10619762; doi:10.3389/fpubh.2023.1185341)
Supplement: Supplementary file 1 [file Data_Sheet_1.pdf]

Supplementary Material

Access to health care for migrants in French Guiana in 2022: a qualitative study of health care system actors

Gabriel Brun-Rambaud*, Leslie Alcouffe, Marc-Alexandre Tareau, Antoine Adenis, Nicolas Vignier

*** Correspondence:** Gabriel Brun-Rambaud: gabriel.brunrambaud@outlook.com

1.1 Supplementary Figures

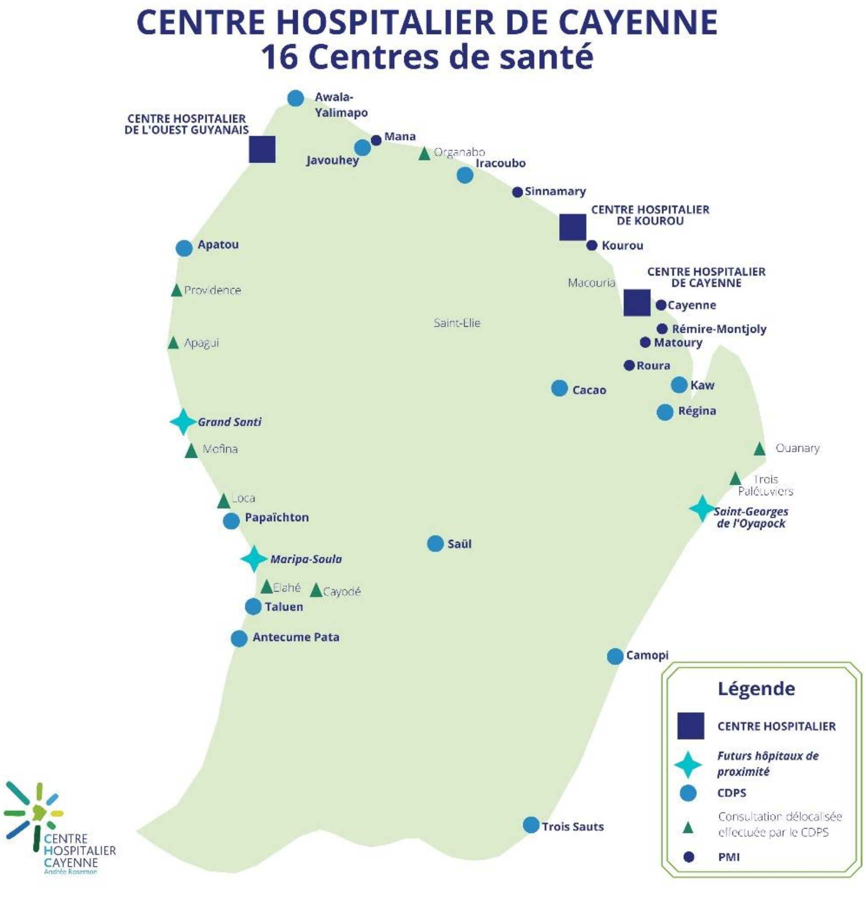

Supplementary Figure 1: Distribution of health structures in French Guiana, GHT, 2022. Translation of the map legend, from top to bottom: *Hospitals, future local hospitals, off-site prevention and health care center (CDPS), off-site consultation provided by CDPS, mother and child protection center (PMI).* Source: Adapted from Groupement Hospitalier de Territoire de Guyane (GHT).

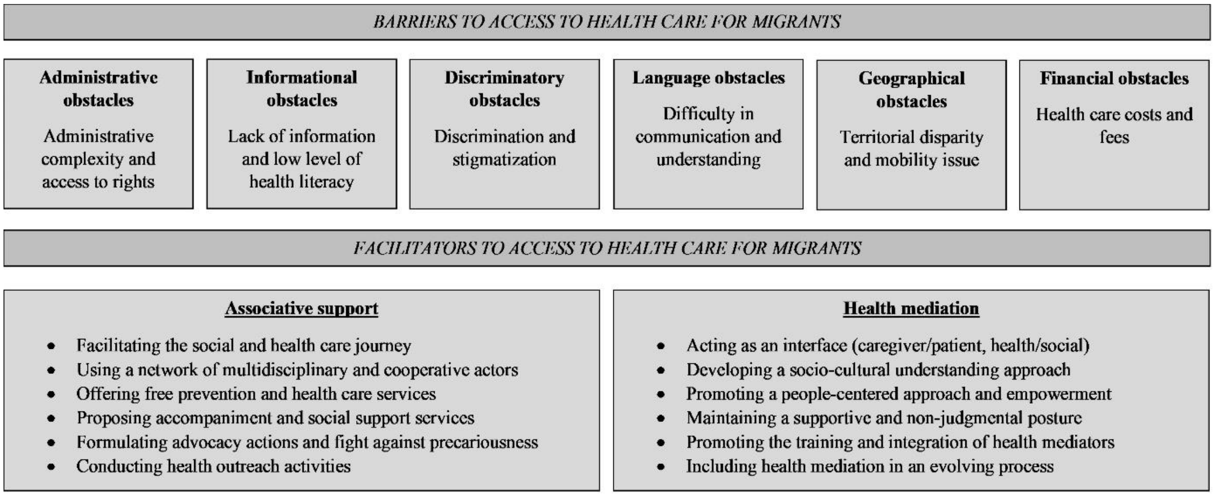

Supplementary Figure 2: Diagram of the main barriers and facilitators to access to health care for migrants reported by health care professionals, social workers and local NGO actors in French Guiana in 2022.
